# Advancing sustainable implementation of an evidence-based mental health intervention in Sierra Leone’s schools: protocol for a hybrid type 3 implementation-effectiveness trial

**DOI:** 10.1186/s12889-024-17928-w

**Published:** 2024-02-03

**Authors:** Alethea Desrosiers, Bidemi Carrol, Haley Ritsema, Walker Higgins, Fatoma Momoh, Theresa S. Betancourt

**Affiliations:** 1 Department of Psychiatry and Human Behavior, Brown University, Warren Alport Medical School, 345 Blackstone Blvd Providence, Providence, RI 02906 USA; 2 RTI International, 701 13th St NW #750, Washington, DC 20005 USA; 3 Innovations For Poverty Action, 47A&B Johnson Street, Freetown, Sierra Leone; 4 Boston College School of Social Work, 140 Commonwealth Avenue, Chestnut Hill, MA 02496 USA

**Keywords:** Implementation science, Mental health, Youth, Low-resource settings

## Abstract

**Background:**

Mental health disorders among youth contribute substantially to the global burden of disease, which is exacerbated in low- and middle-income countries (LMICs) due to large mental health treatment gaps. In Sierra Leone, a West African country with a long history of complex adversity, the mental health treatment gap is estimated at 98%. Implementing innovative mental health interventions that can be sustained at scale is a priority. The Youth Readiness Intervention (YRI) is an evidence-based mental health intervention for youth that can be delivered feasibly by lay health workers/nonspecialists. Using mobile-based technologies to assist implementation could improve the reach and sustainability of the YRI in Sierra Leone. This study aims to train teachers to deliver the YRI in Sierra Leone’s secondary schools and test the feasibility, acceptability, cost, and fidelity to the YRI of a mobile-based supervision model compared with standard, in-person supervision.

**Methods:**

We will conduct a hybrid type 3 implementation-effectiveness cluster randomized trial to assess the feasibility, acceptability, costs and fidelity to the YRI implemented by teachers receiving mobile-based supervision vs. standard supervision. Enrolled schools (*N* = 50) will be randomized to YRI + mobile supervision (*N* = 20), YRI + standard supervision (*N* = 20) or waitlist control (*N* = 10). We will recruit and enroll four teachers per intervention-condition school (*N* = 160) and 1200 youth. We will collect data on implementation outcomes among teachers, principals and youth via a mixed methods approach at baseline and post-intervention. We will also collect quantitative data on youth mental health and functioning as secondary outcomes at baseline and post-intervention, as well as cost-effectiveness data at 12-month follow-up.

**Discussion:**

Study findings have the potential to expand the reach of mental health services among youth in low-resource settings via a teacher workforce. The use of mobile tools, if successful, could support further scale out and sustainment of the YRI to other regions of Sierra Leone and West Africa more broadly, which could help address the mental health treatment gap.

**Trial registration:**

Clinical Trial Network: NCT05737667.

**Supplementary Information:**

The online version contains supplementary material available at 10.1186/s12889-024-17928-w.

## Background

The global burden of disease attributed to mental health disorders among youth and adults is compounded in low-and middle-income countries (LMICs) and other low resource settings due to the wide mental health treatment gap. This is especially problematic in LMICs with a history of violence and loss [1–3]. In Sierra Leone, a country in West Africa that has experienced a long history of compounded adversity (i.e., civil war, the Ebola epidemic), the mental health treatment gap is estimated at 98%, with 49.4 disability adjusted life years per thousand population lost among youth aged 15–29 due to mental health and substance use disorders [4]. Significant limitations in the nation’s health infrastructure have made it difficult to effectively address the population’s unmet mental health needs [4–6]. Given both the limited healthcare infrastructure in Sierra Leone and the high level of mental health needs among youth, identifying alternative delivery settings in which evidence-based mental health interventions can be feasibly delivered by lay workers—both with quality and at sustainable scale—is a priority.

The education sector is a strong option to address the mental health needs of the burgeoning youth population in Sierra Leone by leveraging the teacher workforce and providing strong supervision and training for delivery of evidence-based mental health interventions in schools [7–9]. Prior research in Sub-Saharan Africa supports the feasibility and efficacy of task-shifting strategies that involve teachers as delivery agents to address human resource barriers to mental health service delivery [10–13]. Exploring schools as an alternative delivery setting for evidence-based mental health interventions delivered by a teacher workforce could improve the reach of mental health services for underserved youth and the ability of youth to actualize educational and employment opportunities [14–16]. This also aligns with the priorities of the Government of Sierra Leone to invest in human capital through empowering their youth population (61% of Sierra Leoneans are under age 25) and including student mental health and wellbeing as a strategic priority in their Education Sector plan [8].

**Fig. 1:**
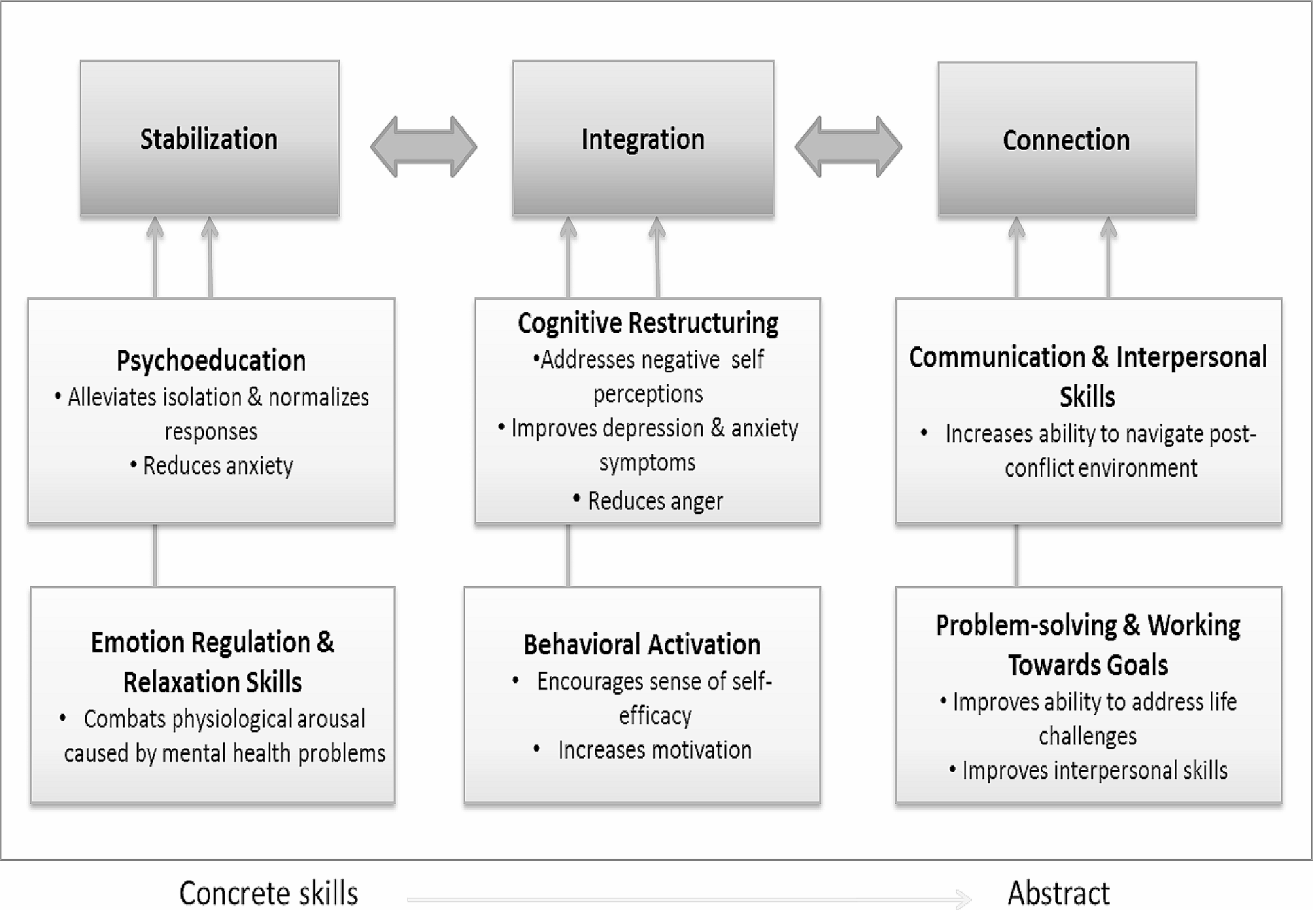
YRI Conceptual Model

In prior research responding to the lack of mental health services in Sierra Leone, a transdiagnostic, common elements-based intervention, the *Youth Readiness Intervention* (YRI), was developed using community-based research methods to promote the mental health of Sierra Leonean youth who had been affected by violence and adversity [17]. The YRI integrates core components of cognitive behavioral therapy, interpersonal therapy, and mindfulness-based techniques and focuses on improving problems with emotion regulation, interpersonal deficits, and problem-solving skills (See Fig. 1) [17, 18]. The YRI has demonstrated effectiveness in improving mental health and daily functioning among vulnerable youth (aged 15–24) enrolled in an alternative education program and was feasibly delivered with quality by lay health workers [18]. Youth who received the YRI exhibited better classroom behavior and were six times more likely than control youth to persist in school. Data from both a pilot study and larger hybrid-implementation-effectiveness trial on the YRI delivered to youth enrolled in an employment promotion program also support the YRI’s mental health benefits [19–21].

The YRI is a promising and scalable approach to targeting underlying risk factors (e.g., poor emotion regulation) related to the onset of mental health problems among youth. Its demonstrated effectiveness in prior research and its simple, transdiagnostic elements make it highly suitable for delivery in resource-constrained community settings (i.e., schools in Sierra Leone) by a range of lay workers with a basic level of education. However, as with other evidence-based mental health interventions in LMICs, the YRI requires innovative implementation strategies for effective scale out and sustainment. Mobile-based strategies to build service delivery capacity and monitor intervention fidelity, particularly those that leverage widely available free commercial applications, have the potential to reduce long-term fiscal and human labor costs associated with traditional modes of supervision in person or via phone; thus, enabling greater scalability in settings with limited behavioral health professionals [22–24]. Applying mobile tools to build teachers’ ability to deliver the YRI’s evidence-based components with fidelity and completence could help decrease the added burdens associated with traditional modes of supervision (e.g., transportation, time) and improve the ultimate sustainability of the YRI within schools in Sierra Leone.

### Study aims

This study will implement the evidence-based YRI as an extracurricular resilience-building activity for youth in Sierra Leone’s secondary schools and train teachers to deliver the intervention. Teachers will receive either mobile phone-based supervision or standard in-person supervision to deliver the YRI. Mobile-based supervision will integrate WhatsApp with an mHealth app designed to support fidelity monitoring and feedback during supervision. Study aims are to: (a) examine the feasibility, acceptability, cost, and fidelity to the YRI delivered by teachers receiving mobile-based supervision compared with those receiving standard supervision; (b) compare the effectiveness of the YRI delivered in schools by teachers who receive either mobile-based supervision or standard supervision; (c) investigate potential mechanisms of YRI adoption and sustainment within schools, including readiness to change and organizational climate; (d) conduct a cost-effectiveness analysis to evaluate the relative costs vs. benefits of the YRI delivered in secondary schools with mobile-based supervision and standard supervision from a broad societal perspective, including educational outcomes.

We hypothesize that: (a) mobile-based supervision will be more feasible, acceptable, and cost-effective than standard supervision, and YRI fidelity scores for mobile supervision will be comparable to standard supervision; (b) youth mental health, emotion regulation, and daily functioning outcomes in mobile supervision will not differ from those in standard supervision over time; (c) readiness to change, organizational climate, and teacher buy-in will moderate YRI adoption and sustainment; (d) the YRI incremental cost-effectiveness ratio in both conditions will surpass the standard willingness to pay threshold [16].

### Study design

We will conduct a hybrid type 3 implementation-effectiveness cluster-randomized trial design [25] to investigate mobile-based supervision of YRI delivery by teachers in Sierra Leone’s secondary schools as a new implementation strategy, with clinical outcomes of the YRI on youth mental and behavioral health as secondary outcomes. We will use a mixed methods approach to investigate study aims. The Exploration, Preparation, Implementation, Sustainment (EPIS) framework [26], a broad, context sensitive implementation science process model, will guide study implementation. Randomization to study condition will occur at the level of the school. Schools (*n* = 50) will be randomized to YRI with mobile-based supervision (*n* = 20 schools/*n* = 480 youth), YRI with standard (in-person) supervision (*n* = 20 schools/*n* = 480 youth) or control (*n* = 10 schools/*n* = 240 youth). Control schools are necessary primarily for the cost-effectiveness analysis because they will provide reference level estimates of QALYs gained by intervention schools. We selected an unequal randomization design to reduce the number of youths who are exposed to the waitlist control [27]. We will offer YRI training to interested teachers in control schools following 12-month follow-up data collection. To optimize comparability between schools, we will match schools into quintets using an algorithm prior to randomly assigning two members of each quintet to each supervision model and the fifth to the control. All research assistants and data analysts will be blinded to participant assignment and will assign schools to study condition based on the allocation sequence.

**Fig. 2:**
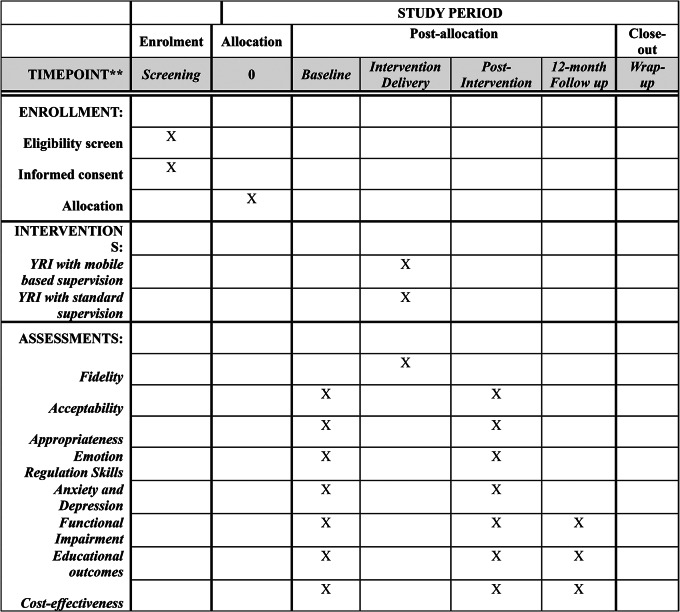
Spirit checklist for the schedule of enrollment, interventions, and assessments

Regarding standard reporting, this study will adhere to the Standard Protocol Items: Recommendations for Intervention Trials (SPIRIT) guidelines (See Fig. 2) and the Consolidated Standards of Reporting Trials (CONSORT). This trial is registered with the Clinical Trials Registry maintained by the National Library of Medicine at the National Institute of Health (Trial ID: NCT05737667; registered on 02/02/23). This study is approved by the Brown University Institutional Review Board (Protocol #2,022,003,442) and the Sierra Leone Ethics and Scientific Review Committee. Any subsequent modifications will be reviewed by both of these review committees for approval and then submitted to the Clinical Trials Registry as an amendment. The study protocol was also eviewed as part of the funding application.

### Intervention description

The YRI is a culturally informed, 12-session, transdiagnostic group intervention that integrates core components of cognitive behavioral and interpersonal therapies as well as mindfulness techniques (i.e., psychoeducation, cognitive restructuring, behavioral activation, interpersonal effectiveness, problem-solving). Sessions typically last approximately 90 min each and incorporate both psychoeducation as well as activities and home practice to hone skills learned. The main objectives of the YRI are to improve emotion regulation, adaptive coping, and interpersonal skills (See Table 1). In the current study, the YRI will be delivered once per week during a time identified by schools when teachers and youth are available.

**Table 1:** YRI Session Modules

Module	Components
1. Introductions and building group cohesion	• Introduces the format and goals of the intervention • Provides an opportunity to begin building trust, motivation for group participation, and group cohesion • Participants identify personal goals that group participation will help them achieve
2. Psychoeducation on Stress and Emotions	• Psychoeducation on traumatic stress reactions in order to normalize this experience for group participants • Participants learn about emotions, how to identify them and how this skill will help them reach their goals
3. Understanding the link between beliefs, bodies and behaviors	• Participants learn about the link between what you think, how it makes you feel, and how you behave • Participants identify healthy and unhealthy coping behaviors and discuss the impact of coping skills on reaching their goals
4. Taking control of your life	• Introduces concept of emotion-regulation and coping skills as one way of taking charge of your life • Practicing of coping strategies and recognizing things you can change and things you cannot • Processing loss and teaching emotion regulation strategies: behavioral activation and deep belly breathing
5. Relaxation and emotion regulation	• Participants reflect upon what types of behaviors can alter their mood for the better • Review of belly breathing and learn about guided imagery (safe spaces) • Participants identify fun activities that can disrupt negative thinking
6. Dealing with the past, things lost/things gained	• Focus on normative reactions to life disruption and loss related to traumatic experiences and loss • Participants reflect on things lost but also things gained in their lives
7. Sequential problem solving	• Participants learn how to approach decision-making using sequential (step-by-step problem-solving) • Practicing of sequential problem solving
9. Review of coping skills and problem solving	• Participants review and practice skills learned thus far • Participants learn another relaxation strategy: progressive muscle relaxation • Participants discuss use of the skills to attain their goals.
10. Addressing negative self-perceptions	• Interactive exercises are used show how negative beliefs affect their emotions and their behaviors • Participants learn strategies for changing their focus to the positive, including distraction by engaging in a fun or relaxing activity
11. Review of skills and relapse prevention	• Review of the skills learned in the group and planning for future challenges • The group will think about challenges in the future, review their warning signs for when they begin to feel upset, and discuss the strategies they have learned to help themselves feel better
12. Celebration and moving forward	• Reinforcing the important concepts mastered throughout the group process • Participants reflect on the information that they felt impacted them the most. • Participants have an opportunity to express appreciation of the things they have learned • Celebrations in the community are an optional component

### mHealth app development for mobile supervision

Drawing from best practices in user-centered design [28–30] and lessons learned from a prior pilot trial of an mHealth quality improvement app in Sierra Leone (NIMH MH124071), we are completing development of the mHealth supervision app that will also integrate WhatsApp as a communication platform. During an initial problem analysis phase, we convened one focus group discussion with teachers in Sierra Leone (*N* = 8) and one with YRI experts (*N* = 4) to determine how best to use WhatsApp to complement mHealth tools to facilitate supervision and fidelity monitoring. Focus groups sought to understand potential forms of 1- and 2-way communication, must-have functions to improve mHealth tools, how WhatsApp and mHealth tools can work together, and potential challenges of the mobile-based supervision model. We have incorporated information from the focus groups into design and development of an initial prototype. We will complete two rounds of iterative user experience/user interface testing with focus group participants to elicit “end-user” feedback while using mHealth tools prior to launching the clinical trial. Feedback from user testing sessions will inform final refinements of the integrated mobile supervision model.

### Settings and participants

We will recruit 50 schools from the Western region of Sierra Leone to participate in the hybrid type 3 implementation-effectiveness cluster randomized trial. The Western region of Sierra Leone comprises two districts: Western Urban and Western Rural. The capital city of Freetown is located in the urban area of the Western region. English is the official language, and Krio is the lingua franca. According to the Sierra Leone Demographic and Health Survey in 2019 data on educational attainment in the Western Area, over 55% of men and 48% women have attained some secondary education. According to the 2020 Annual School Census report, there are 240 senior secondary schools in the Western Area (77 in Western Rural and 163 in Western Urban), which account for over a third (36%) of all senior secondary schools in the country. Most schools are co-educational, and just over half are public schools.

We will aim for balance in recruitment of schools from urban and rural areas. Potential schools in the Western region will be identified through publicly available databases maintained by the Government of Sierra Leone’s Ministry of Basic and Senior Secondary Education (MBSSE). Trained research assistants (RAs) (who have completed CITI training and training in good clinical practice) will use recruitment scripts to explain the study to eligible participants and determine if they are interested in participating. School eligibility criteria are: (a) a secondary school in the Western region that is (b) willing to provide the YRI as an extracurricular activity. RAs will first recruit and enroll principals (*N* = 50). RAs will obtain informed consent from interested principals of eligible secondary schools.

Principals who consent and enroll will then provide lists of teachers in their school. RAs will recruit four teachers per enrolled school (*N* = 160; 80 female/80 male). Teacher inclusion criteria are (a) currently employed as a teacher in a secondary school in the Western Region; and (b) willing to provide the YRI as an extracurricular activity. RAs will aim for gender balanced recruitment of teachers from enrolled schools because the YRI is delivered by gender-matched facilitators. RAs will contact teachers to describe the study and invite them to participate. RAs will obtain informed consent from interested teachers who meet eligibility criteria.

We will recruit 480 youth from the 40 secondary schools randomized to receive the YRI delivered with mobile supervision and 480 youth from schools that will receive YRI delivered with standard supervision. We will enroll 240 youth from 10 control schools. We will aim to recruit 24 youth per school, with two YRI groups per intervention school. We aim to recruit an equal number of male and female participants, as the YRI is delivered in separate groups for males and females. The target sample size of 1200 youth is powered to account for 10–20% attrition per our prior experience in Sierra Leone. Youth inclusion criteria are: (a) currently enrolled in a secondary school in the Western region; (b) male or female aged 14–24; (c) able and willing to attend an extracurricular school activity. Parental consent and youth assent will be obtained for all youth under age 18.

Our target age for enrollment of youth (aged 14–24) is based on prior research on the YRI demonstrating that the intervention is effective with male and female populations within this age range [19, 20]. Including students 18–24 reduces potential bias towards those youth who take longer to complete secondary school, delayed entry into school, or dropped out of school and later returned. Excluding students older than age 24 ensures we are targeting the developmental window of youth as defined by the United Nations and the World Health Organization.

### YRI training and supervision

Following a train-the-trainer approach, we have hired a cadre of local experts previously trained in the YRI (*n* = 12) to conduct the two-week (10-day), in person YRI training with teachers who will deliver the YRI. Training will feature role playing, didactics, direct practice with the YRI manual, and group discussion. YRI experts will directly observe role plays of session modules and use the YRI fidelity checklist, a tool developed and tested in previous YRI trials, to rate fidelity to session content as well as competency during role plays and provide feedback. YRI trainees (teachers) will also complete a competency assessment at the end of training. Scores of > 70% will indicate YRI competency. For teachers scoring < 70%, YRI experts will provide “booster” training sessions targeting weakness identified in the competency assessment. Ten of the 12 YRI experts who deliver training will also serve as supervisors during the implementation phase. YRI supervisors and facilitators will complete a 1-day technology training led by the app development team and the Program Manager to address potential technology literacy challenges. YRI supervisors and facilitators will explore key features of the mHealth supervision app and WhatsApp through “hands on” testing/discovery and role-plays. The Program Manager will also review basic mobile phone functions and help troubleshoot any issues that arise.

YRI facilitators (teachers) in both study conditions will complete the fidelity and competency checklists after each session and audio record all sessions. Given the potential bias of self-report, supervisors will also code randomly selected 20-minute segments after each session. YRI fidelity checklists include both cross-cutting session items (e.g., encourages participation) to assess competency and session-specific items (e.g., facilitator reviewed deep belly breathing) to assess fidelity to content. Mobile-based supervision will include a designated 30–45 min weekly “supervision chat” using WhatsApp, review of audio taped session segments and fidelity and competency checklist data, as well as options for asynchronous messaging and chats as needed to provide targeted and supportive feedback, share lessons learned, and answer questions. Standard supervision will occur via weekly 30–45 min in-person sessions and include review and discussion of fidelity data. YRI facilitators who receive mobile-based supervision will complete the YRI fidelity and competency checklists as an electronic fillable form embedded in the mHealth app; those who receive standard supervision will complete a manual checklist in Excel uploaded to a secure server (Box).

#### Data collection

Data collection will be at three levels: teacher, principal and youth. Teachers (*N* = 160) and principals (*N* = 40) will complete quantitative assessments on implementation-level outcomes at baseline and post-intervention. All principals and a subset of teachers (*N* = 40) will also complete qualitative exit interviews at post-intervention to explore key aspects of feasibility, acceptability, and adoption, (e.g., usefulness of YRI skill-building exercises), and internal and external factors that might influence facilitator experiences (e.g., school climate, incentives). The subset of teachers will be selected based on a multivariate sampling matrix [31, 32]. We will also explore feasibility and acceptability of mobile-based supervision via key informant interviews with facilitators who received mobile supervision at post-intervention.

Youth (*N* = 1200) will complete quantitative assessments on mental health and daily functioning at baseline and post-intervention. Youth from intervention schools (*N* = 960) will complete quantitative assessments on implementation-level outcomes. A subset of youth (from intervention schools; *N* = 32) randomly selected based on a multivariate sampling matrix will complete exit interviews at post-intervention to obtain their feedback on YRI feasibility, acceptability, and barriers/facilitators to adoption. We will also collect data on educational outcomes for all youth via administrative records (i.e., school attendance, grades, tardiness, disciplinary reports) at baseline, post-intervention and 12-month follow-up. All youth will complete the EQ-5D-3 L at baseline, post-intervention and 12-month follow-up to inform the cost-effectiveness analysis [33].

Trained RAs will administer assessments electronically via tablets. All classroom instruction, assignments and tests are completed in English in our catchment area, thus the Western region schools, assessments and interviews will be completed in English as a first option. Versions of all study assessments will also be available in Krio (the local language) for any participants indicating difficulties with English fluency or a preference for completing assessments in Krio. All measures have been forward and backward translated from English to Krio previously. We will report data disaggregated by sex.

### Measures

#### Implementation outcomes

Feasibility, acceptability, appropriateness, adoption, sustainment, and organizational climate will be assessed via the Implementation Science Questionnaire developed at Johns Hopkins Bloomberg School of Public Health [34]. We have used these measures in prior research in Sierra Leone [20, 21], and the scales demonstrated strong psychometric properties (α = 0.79 − 0.90). The measures will be administered to youth, teachers and principals at baseline and post-intervention. Readiness for Implementing Change will assess readiness to change [35]. This measure has been used cross-culturally and has demonstrated strong psychometric properties (α = 0.94). These core constructs will also be probed via post-intervention key informant interviews with youth, teachers, and principals. We will administer the System Usability scale at post-intervention to assess mHealth app usability [36]. Fidelity will be assessed using the YRI fidelity checklists completed either via an electronic fillable form in the app (mobile supervision condition) or using Excel spreadsheets (standard supervision).

#### Youth mental health outcomes

The Difficulties in Emotion Regulation Scale (DERS), will assess emotion regulation skills [37]. The DERS is a 36-item scale scored on a 5-point Likert scale, with higher scores reflecting greater difficulties regulating emotions (α = 0.91). The Hopkins Symptom Checklist (HSCL) will measure symptoms of anxiety and depression [38, 39]. The HSCL-25 consists of ten items scored on a 4-point Likert scale for anxiety symptoms (α = 0.82) and 15 for depression symptoms (α = 0.82), with higher scores reflecting worse functioning. The Adapted Youth Risk Behavior Survey is a 14-item scale adapted from the original 89-item scale that will assess priority health risk behaviors among youth (i.e., behaviors that contribute to unintentional injuries and violence, tobacco use, alcohol and other drug use, sexual behaviors that contribute to unintentional pregnancy and sexually transmitted infections) [40, 41]. Items include both binary (yes/no) and multiple response options. The World Health Organization Disability Assessment Schedule (WHO-DAS)-2.0 short form will assess functional impairment [42]. The WHO-DAS short form consists of 12-items measuring functional impairment across 5 domains: mobility, self-care, understanding and communication, life activities and societal participation. Items are scored on a 5-point Likert scale and summed to derive a total scale. Higher scores indicate worse functioning [40]. The EuroQual-5 Dimension (EQ-5D-3 L) will be used to calculate quality adjusted life years (QALYs) among youth for the cost-effectiveness analysis [43]. The EQ-5D-3 L is designed for describing and valuing health in terms of 5 dimensions: mobility, self-care, usual activities, pain/discomfort, and anxiety/depression [33]. Each dimension has 3 response categories rated as “no problems”, “some problems”, or “extreme problems”.

### Data analysis

#### Quantitative analysis

We will compare fidelity trajectories of teachers receiving mobile supervision to those of teachers receiving standard supervision and investigate the null hypothesis that mobile supervision will not differ from standard supervision. We will conduct exploratory analyses to uncover typical trajectories for each supervision method (mobile-based or standard) and undertake comparisons using growth curve modeling by means of multilevel models [44]. We will use HLM software (v.8.2), which estimates multiple imputations based on the dependencies of multilevel data. Missing time points are addressed by the multilevel approach to growth modeling, and data from all enrolled participants can be analyzed in accordance with our intention to treat design. The null hypothesis is that the outcomes for the mobile supervision YRI groups will not differ from those in standard supervision YRI groups. With this approach, we can compare both YRI models (as identified by dummy variables) to the untreated control group or assess the null hypothesis of no difference between the fidelity in the two models (mobile-based vs. standard supervision).

We will also investigate the null hypothesis that mobile supervision will not differ from standard supervision at post-intervention in youth outcomes. To investigate YRI effects on mental health among youth at post-intervention, we will use multilevel models to accommodate the clustering of students within YRI groups. For analysis of student outcomes, student characteristics will be modeled in a level-one equation. The group characteristics will be modeled in the level-two equation. School characteristics will be incorporated into a third level.

However, we do not expect a significant school-level intraclass correlation (ICC) for targeted YRI outcomes. In the event we do not find a significant ICC, sector and rural/urban will be represented in the group-level equation, and a two-level model will be estimated.

#### Statistical power analysis for fidelity to YRI implementation

To assess the minimal detectable effect size for the two-tailed null hypothesis of no difference for fidelity of groups receiving mobile versus standard supervision requires an estimate of the within-group ICC for fidelity for which there is no clear guidance in the literature. Given our standard assumptions, at a small to medium ICC of 0.2 for a two-tailed test with a null hypothesis of no difference for 80 groups and 12 fidelity ratings, the minimum detectable effect size would be 0.30, while at a large ICC of 0.5 the minimum detectable effect size would be 0.41.

#### Statistical power analysis for student outcomes

Power will be sufficient to assess our secondary aim and examine the hypothesis that the YRI delivered by teachers participating in mobile supervision will be noninferior in terms of youth mental health outcomes compared to the YRI delivered by teachers receiving standard supervision. At a power of 0.8 and an alpha of *p* <.05 (one-tailed to assess noninferiority), using an ICC of 0.03 (based on prior ICCs for mental health outcomes of 0.01-0.04) [18] for the group for comparisons at a single time point (post-intervention or 12-month follow-up) for student outcomes, the minimal detectable standardized effect size for a standardized difference (d) between the two of 0.21; in other words, at a negative difference of 0.21 or smaller, mobile supervision will be considered noninferior to standard. To test individual null hypotheses that either YRI implementation model (20 schools per YRI supervision mode) is superior (one-tailed) to the 10 untreated control schools with regard to mental health or educational outcomes, we have 0.8 power to detect a standardized effect of 0.26. In a comparison of all 40 YRI schools as compared to the 10 control schools, the minimum detectable effect would be 0.21.

#### Qualitative data analysis

We will use an analytical strategy based on thematic content analysis to analyze qualitative data [45, 46]. Audio-recorded qualitative data, transcribed and with identifying information removed, will be analyzed using Nvivo. We will subject all data to an coding process guided by the Proctor Implementation Outcomes framework [47]. We will identify key themes and iteratively develop a coding scheme, organized according to key themes (i.e., barriers and facilitators to YRI delivery) and map key themes onto the Proctor Framework (i.e., feasibility, acceptability, adoption, appropriateness, sustainment) [47]. Two team members trained in coding scheme will independently code 10% of transcripts to examine reliability. Poor agreement (i.e., low kappa ratings as scored in the qualitative analysis program Nvivo) will be grounds for refining the coding scheme or retraining. Once all coding schemes demonstrate high reliability, team members will code the full qualitative dataset in Nvivo using the robust code books. Qualitative and quantitative implementation outcomes data will be synthesized and triangulated to understand barriers and facilitators to digital platform implementation as well as factors influencing adoption and “buy-in” of the platform within schools. We will integrate data from qualitative and quantitative sources using mixed methods “joint displays” to identify areas of convergence/divergence.

#### Cost-effectiveness analysis

We will conduct a cost-effectiveness analysis from a broad, societal perspective to estimate the social cost per QALY gained [24, 48]. Social cost factors all health costs stemming from an intervention, including the value of participants’ time, and is recommended by the Second Panel on Cost-Effectiveness in Health and Medicine for use in economic evaluations [49]. To estimate costs, we will use an ingredients approach in which activities are first identified as costs, and then the financial and economic costs of carrying out these activities are quantified [48]. Financial costs account for actual monetary expenses while economic costs encompass all resources, including those volunteered, and opportunity costs incurred by participants. Activities include staff and interventionist trainings, participant recruitment, session delivery, and review of work with supervisors. We will get cost estimates from a range of sources (e.g., invoices, receipts, budgets from implementation partners); staff time will be costed at full salary. All costs will be appropriately prorated to reflect actual effort or “real time spent”. We will request expenditure reports from partners to double check the ingredients-based costing approach and triangulate cost estimates. We will calculate the time invested by each YRI participant to derive a value of this time in dollars, based on median wages in Sierra Leone. All costs will be expressed in US dollars.

We will derive QALYs gained from the mental health impacts of the YRI supported by mobile supervision and standard supervision, measured using the EQ-5D-3L. We will convert EQ-5D-3L scores to QALYs. To generate QALYs from the EQ-5D-3L, we will calculate a summary index using a standard and relevant weighting scheme. In our case, the index scores will be converted to QALYs using weights developed for Zimbabwe [49]. We will track EQ-5D-3L scores across the duration of the study and derive the QALY difference between YRI with mobile supervision, YRI with standard supervision and control participants—an approach validated in previous studies. For the intervention to be cost-effective, each QALY gained must cost less than three times Sierra Leone’s gross domestic product at purchasing power parity [16, 50]; this follows standards for good value for money outlined in global health interventions [33, 51]. To extend our analysis beyond health to education, we will also estimate the cost per additional year of school enrollment.

### Dissemination

Study leaders are committed to the timely dissemination of research outcomes. The data generated in this study will be presented at national and/or international conferences and published in a timely fashion. All final peer-reviewed manuscripts that arise from this proposal will be submitted to the digital archive PubMed Central. For data that is de-identifiable, we will make data available in accordance with the National Institute of Health guidelines and the IRB.

Our study team will also explore multi-pronged strategies to disseminate findings, such as policy briefs, newspaper articles, radio announcements, and social-media site postings of study findings to target multiple audiences through multiple modalities, including key stakeholders and policy makers (e.g., Ministry of Basic and Senior Secondary Education, Ministry of Youth Affairs). We will provide stakeholders with resources to support adoption and sustainment of the YRI aided by mobile-based supervision after we complete our evaluation, such as mHealth app user resources and evidence briefs that can be shared with government stakeholders across ministries.

## Discussion

This study seeks to conduct a hybrid type 3 implementation-effectiveness cluster randomized trial to evaluate a new implementation strategy: mobile-based supervision for teachers delivering an evidence-based mental health intervention in Sierra Leone’s secondary schools and compare this strategy with standard, in-person supervision for teachers delivering the intervention. Research findings have the potential to expand the reach of evidence-based mental health services through a teacher workforce and open-source platforms. Training teachers to deliver evidence-based mental health interventions within public and open government subsidized schools has the potential to reach a larger population of vulnerable youth. Leveraging low-cost technology can improve the efficiency, effectiveness, and scalability of evidence-based mental health services in Sierra Leone as well as in other LMICs and resource-constrained settings and help offset the financial and human resource burdens associated with traditional modes of performance monitoring and quality improvement cycles. The mobile tools, if feasible and acceptable, could be modified to support teacher training, teaching quality, and adherence to the school curriculum. They also have the potential to support further scale-out and sustainment of the YRI throughout schools across other regions of Sierra Leone and West Africa more broadly, which could ultimately help to reduce the mental health treatment gap in this region.

Despite the potential cost-effectiveness results that we anticipate from using mobile-app supervision, sustainability remains a persistent challenge for implementation in LMICs due to limited financial, human and material resources. This could be addressed by engaging multiple stakeholders from the grassroots to the government level. We plan to engage the MBSSE, Sierra Leone’s Teaching Service Commission, and both in-country and international nongovernmental organizations to determine how best to sustain the program afterresearch funding has ended. Via these conversations, we hope to collaborate in co-creating a strategy to support YRI sustainability and to facilitate the capacity building necessary to bridge implementation gaps and increase sustainment of evidence-based practice.

### Trial status

At the time of manuscript submission, we had recruited and enrolled principal and teacher participants. Preparations were underway to begin youth participant recruitment.

### Electronic supplementary material

Below is the link to the electronic supplementary material.

Supplementary Material 1: SPIRIT 2013 Checklist: Recommended items to address in a clinical trial protocol and related documents*

## Data Availability

Not applicable; Data collection described in this study protocol has not started.

## Abbreviations

LMIC: Low-and middle-income country
YRI: Youth Readiness Intervention
MBSSE: Ministry of Basic and Senior Secondary Education
RA: research assistant
QALY: Quality adjusted life years
WHODAS: World Health Organization Disability Assessment Schedule
DERS: Difficulties in Emotion Regulation Scale
HSCL: Hopkins Symptom Checklist
EQ-5d-3L: EuroQual-5 Dimension
ICC: Interclass Correlation
