# Supplementary material for: Advancing sustainable implementation of an evidence-based mental health intervention in Sierra Leone’s schools: protocol for a hybrid type 3 implementation-effectiveness trial
Source: BMC Public Health. 2024 Feb 3;24:362. doi: 10.1186/s12889-024-17928-w (PMC10837990; doi:10.1186/s12889-024-17928-w)
Supplement: Supplementary file 1 — Supplementary Material 1: SPIRIT 2013 Checklist: Recommended items to address in a clinical trial protocol and related documents* [file 12889_2024_17928_MOESM1_ESM.docx]

SPIRIT 2013 Checklist: Recommended items to address in a clinical trial protocol and related documents*

| Section/item | ItemNo | Description |
| --- | --- | --- |
| **Administrative information** | | |
| Title | 1 | Advancing mHealth-supported Adoption and Sustainment of an Evidence-based Mental Health Intervention for Youth in a School-based Delivery Setting in Sierra Leone |
| Trial registration | 2a | Clinical Trials Registry maintained by the National Library of Medicine at the National Institute of Health; Trial ID: NCT05737667 |
|  | 2b | N/A |
| Protocol version | 3 | Protocol Version 1; Brown University IRB Protocol Identifier #2022003442; 12/22/22 |
| Funding | 4 | National Institute of Mental Health; R01 MH130320 |
| Roles and responsibilities | 5a | AD [Brown University]; BC [RTI]; HR [Brown University]; TSB [Boston College]; WH [Innovations for Poverty Action]; FM [Innovations for Poverty Action]  AD made a substantial contribution to the conception and design of the work, interpretation of the data for the manuscript, and drafting the work. BC, HR, and TSB made substantial contributions to interpretation of the data for the manuscript and drafting the work. WH and FM made substantial contributions to data acquisition. All authors contributed to drafting and/or revising the manuscript for important intellectual content; provided final approval of the version to be published; and agree to be accountable for all aspects of the work. |
|  | 5b | This trial is sponsored by Brown University, Biomed Research Administration; 222 Richmond Street, Providence, RI -2903; 401-863-1000. |
|  | 5c | Neither the study funder nor sponsor will play any role in the design of the study, collection of study data, analysis of study data, interpretation of findings, writing of the report, and decisions to submit results. |
|  | 5d | This trial will be overseen by an independent study monitor |

| Introduction |  |  |
| --- | --- | --- |
| Background and rationale | 6a | Mental health disorders are the second-largest contributor to the global burden of disease among youth and adults. The prevalence of untreated mental disorders among adults in LMICs may be as high as 78%; for adolescents and youth, the figure is likely higher. In Sierra Leone, the mental health treatment gap is estimated at 98%, with 49.4 disability adjusted life years (DALYs) per thousand population lost among youth aged 15-29 due to mental health and substance use disorders. Given both the limited health infrastructure in Sierra Leone and the high level of unmet mental health needs among youth, identifying alternative delivery settings in which evidence-based mental health interventions can be feasibly delivered by lay workers—both with quality and at sustainable scale—is a priority.  In prior research addressing mental health treatment gaps in Sierra Leone, a cognitive behavioral therapy-based intervention, the *Youth Readiness Intervention* (YRI), demonstrated effectiveness in improving mental health and daily functioning among vulnerable youth (aged 15-24) enrolled in an alternative education program and was feasibly delivered with quality by lay health workers. Youth who received the YRI exhibited better classroom behavior and were six times more likely than control youth to persist in school. Preliminary pilot study data on the YRI delivered to youth enrolled in an employment training program also support the YRI’s mental health benefits. The YRI is a promising and scalable approach to targeting underlying risk factors (e.g., poor emotion regulation) related to the onset of mental health problems. The YRI, as with other evidence-based mental health interventions in LMICs, requires innovative implementation strategies for effective scale up and sustainment. We will implement the evidence-based YRI as an extracurricular resilience-building school activity and provide teachers with intensive mobile phone-based supervision to deliver the YRI. |
|  | 6b | A waitlist control condition is included for the planned cost-effectiveness analysis. The supervision comparisons have been selected to evaluate the benefits of mobile supervision compared with the standard approach. |
| Objectives | 7 | This study will implement the evidence-based YRI delivered by teachers in Sierra Leone’s secondary schools. Study aims are to: (a) examine the feasibility, acceptability, cost, and fidelity to the YRI delivered by teachers receiving mobile-based supervision compared with those receiving standard supervision; (b) compare the effectiveness of the YRI delivered in schools by teachers who receive either mobile-based supervision or standard supervision; (c) investigate potential mechanisms of YRI adoption and sustainment within schools, including readiness to change and organizational climate; (d) conduct a cost-effectiveness analysis to evaluate the relative costs vs benefits of the YRI delivered in secondary schools with mobile-based supervision and standard supervision.  We hypothesize that: (a) mobile-based supervision will be more feasible, acceptable, and cost-effective than standard supervision, and YRI fidelity scores for mobile supervision will be comparable to standard supervision; (b) youth mental health, emotion regulation, and daily functioning outcomes in mobile supervision will not differ from those in standard supervision over time; (c) readiness to change, organizational climate, and teacher buy-in will moderate YRI adoption and sustainment; (d) the YRI incremental cost-effectiveness ratio in both conditions will surpass the standard willingness to pay threshold. |
| Trial design | 8 | The design is a hybrid Type III Implementation-Effectiveness Cluster-Randomized Trial with three arms. Schools are randomized to one of three conditions: (a) YRI delivery with mobile supervision, (b) YRI delivery with standard supervision (in person), (c) waitslist control. |

| Methods: Participants, interventions, and outcomes | | |
| --- | --- | --- |
| Study setting | 9 | This study will take place within secondary schools in Sierra Leone’s Western Region. |
| Eligibility criteria | 10 | Schools / Principals inclusion criteria:   - Secondary school in the Western Region of Sierra Leone - Willing to provide YRI as an extracurricular activity   Youth inclusion criteria:   - Currently enrolled in a secondary school enrolled in the study - Male or female aged 14-24 - Able to attend afterschool activities   Teachers inclusion criteria:   - Currently employed at an 3n4oll3e secondary school - Willing to provide the YRI as an extracurricular activity |
| Interventions | 11a | Mobile-based supervision will include a designated 30-minute weekly “supervision chat,” review of YRI session fidelity data, and options for asynchronous messaging as needed to provide targeted feedback or to answer questions. Visual displays of fidelity data generated in the app will be referred to during supervision sessions to guide discussions and feedback. Standard supervision will occur via weekly 30-45-minute in-person sessions and include review and discussion of fidelity data. Gender-matched teachers who have successfully completed YRI training will deliver the 12 YRI sessions (90 minutes weekly) to male and female student groups with the support of mobile-based or standard supervision. YRI session delivery will not differ in the two groups. More details on YRI content can be found in the study protocol. Teachers in waitlist control schools will be offered YRI training after the 12-month follow-up data collection period has ended. Teachers in waitlist control schools who successfully complete YRI training may elect to provide the YRI to interested students with the permission of their school principal. |
|  | 11b | Participants may choose to withdraw from the study at any time. There are no other modifications to intervention allocation that will occur. |
|  | 11c | Teacher participants will meet weekly with YRI supervisors. During this time, teachers will be reminded of the study protocol. YRI supervisors will meet weekly with the YRI Supervision Team Coordinator and will be reminded of the study protocol during this time. Adherence to data collection procedures will be monitored during biweekly meetings with the data collection team. Reminders will be provided on the study protocol as needed. Teachers will take attendance during each YRI session to monitor youth attendance rates and adherence to session participation. Youth will be contacted who miss more than one session and encouraged to join the next session. Teachers will help youth troubleshoot any barriers to participation. |
|  | 11d | No requirements or restrictions. |
| Outcomes | 12 | Primary Outcomes  1.Fidelity: Fidelity will be measured with the YRI Fidelity Checklist, a tool developed and tested in previous trials that is completed after each YRI session.  [Time Points: For 12 weeks, starting from the date of the first YRI session; The total score for each session’s checklist will be calculated, and scores for each session will be compared between teachers in each supervision condition (i.e., the two active intervention arms)]  2.Acceptability: Acceptability, or the level of satisfaction with the intervention, will be assessed with the Johns Hopkins University Implementation Science Questionnaire. Total scores are calculated [Time Points: Baseline, Post-intervention; Total scores at post-intervention will be compared between participants in each intervention arm]  3.Appropriateness: Appropriateness, or the relevance and fit of the intervention, will be assessed with Johns Hopkins University Implementation Science Questionnaire. [Time Points: Baseline, Post-intervention; Total scores at post-intervention will be compared between participants in each intervention arm]  Secondary Outcomes  1. Emotion regulation skills: Emotion regulation will be measured with the Difficulties in Emotion Regulation Scale. [Time Points: Baseline, Post-Intervention; Change over time in total scores will be compared across study arms]  2. Anxiety and Depression: Anxiety and Depression will be measured with the Hopkins Symptom Checklist, a 25-item inventory that measures symptoms of anxiety and depression. [Time Points: Baseline, Post-Intervention; Change over time in total scores will be compared across study arms]  3. Functional Impairment: Functional Impairment will be measured by the World Health Organization Disability Assessment Schedule short-form. [Time Points: Baseline, Post-Intervention; Change over time in total scores will be compared across study arms] |
| Participant timeline | 13 | Teachers will deliver the 12 YRI sessions once per week. Session delivery will start after all baseline data collection is completed (1-2 weeks). Data collection will occur at baseline and post-intervention for all participants. Youth participants will also complete a brief assessment at 12-month follow-up. School administrative data for educational outcomes will be collected at baseline, post-intervention and 12-month follow-up. A 4-week window will be available to complete data collection at post-intervention and 12-month follow-up. |
| Sample size | 14 | *Statistical power analysis for fidelity to YRI implementation*: To assess the minimal detectable effect size for the two-tailed null hypothesis of no difference for fidelity of groups receiving mobile versus standard supervision requires an estimate of the within-group ICC for fidelity for which there is no clear guidance in the literature. Given our standard assumptions, at a small to medium ICC of 0.2 for a two-tailed test with a null hypothesis of no difference for 80 groups and 12 fidelity ratings, the minimum detectable effect size would be 0.30, while at a large ICC of 0.5 the minimum detectable effect size would be 0.41.  *Statistical power analysis for student outcomes*: At a power of .8 and an alpha of p<.05 (one-tailed to assess noninferiority), using an ICC of .03 (based on prior ICCs for mental health outcomes of .01-.04)^9^ for the group for comparisons at a single time point (post-intervention or 12-month follow-up) for student outcomes, the minimal detectable standardized effect size for a standardized difference (d) between the two of 0.21; in other words, at a negative difference of .21 or smaller, mobile supervision will be considered noninferior to standard. To test individual null hypotheses that either YRI implementation model (20 schools per YRI supervision mode) is superior (one-tailed) to the 10 untreated control schools with regard to mental health or educational outcomes, we have 0.8 power to detect a standardized effect of 0.26. In a comparison of all 40 YRI schools as compared to the 10 control schools, the minimum detectable effect would be 0.21.  Total sample size youth: 1200; Total sample size teachers: 160; Total sample size principals: 40. |
| Recruitment | 15 | Our target sample size of 1200 youth is powered to account for 10-20% attrition per our prior experience in Sierra Leone. Schools census records will be reviewed to ensure that schools selected for recruitment have more than the target number of teachers and students to help ensure that target number, including gender balance where possible, can be reached. |

| **Methods: Assignment of interventions (for controlled trials)** | | |
| --- | --- | --- |
| Allocation: |  |  |
| Sequence generation | 16a | Schools will be randomly assigned to either YRI delivery with mobile supervision (N=20), YRI delivery with standard supervision (N=20), or waitlist control (N=10) study conditions using a computer-generated allocation sequence and an uneven randomization strategy (2:2:1). |
| Allocation concealment mechanism | 16b | Schools will be randomized using the STATA generated randomization sequence. Study RAs will ensure that participants do not have access to the randomization sequence. Participants will not be informed about their study assignment until after they have completed baseline assessments. |
| Implementation | 16c | The allocation sequence will be generated using STATA. All schools in which principals provided informed consent will be randomized to a study condition. All youth and teacher participants who have provided informed consent from the enrolled, randomized schools will participate in the activities associated with the respective study condition assigned to their school. Study RAs will contact participants to inform them of their study condition assignment after they have completed baseline assessments. |
| Blinding (masking) | 17a | Study RAs will be blinded to study condition of participants. Biostatisticians performing data analysis will also be blinded to study condition of participants. |
|  | 17b | Unblinding will be avoided to the greatest extent possible to avoid any potential biases during assessment periods and data analysis. There are no anticipated situations in this study in which unblinding would be necessary. |

| **Methods: Data collection, management, and analysis** | | |
| --- | --- | --- |
| Data collection methods | 18a | Quantitative assessments will be administered via tablets by trained study RAs using the **Survey CTO** platform, which is HIPAA compliant. RAs will complete CITI training and training in research ethics (Good Clinical Practice), including methods to protect participant confidentiality. All quantitative measures have been forward and backward translated following WHO guidelines for adaptation and translation. Measures have demonstrated strong reliability [α=.72-.96]. All youth participants will complete quantitative assessments on mental health and implementation outcomes at baseline (prior to starting the intervention) and post-intervention (after all YRI sessions have been completed). Teacher and principal participants will complete quantitative assessments on implementation outcomes at baseline and post- intervention. A sub-set of youth, teachers, and principals will also participate in qualitative exit interviews. All data collection will take place at a private location either in the participant’s school or at another private location in their community.  Measures: Difficulties in Emotion Regulation- Short form (DERS); WHO Disability Assessment Schedule (WHODAS); Hopkins Symptom Checklist (HSCL); Sierra Leone Demographics and Health Survey-Intimate Partner Subscale; Adapted Youth Risk Behavior Survey (YRBS); EuroQol-5 Dimension (EQ-5D-3L); Feasibility, Acceptability, Adoption, Appropriateness, and Sustainment of the YRI as well as organizational climate will be measured with quantitative scales developed by researchers at Johns Hopkins Bloomberg School of Health; Organizational Readiness for Implementing Change (ORIC; Shea et al., 2014) will assess readiness to change; YRI Fidelity Checklist will assess fidelity to YRI session content and overall competency. |
|  | 18b | To maximize YRI participant completion and retention rates through post-assessment and the 12-month follow-up cost-effectiveness data collection, we will utilize structured activities that are considered core principles in conducting longitudinal studies and have been successful in many different cultural settings. RAs will maintain accurate and timely contact information for all participants throughout the study. This electronic contact file will be incorporated into SurveyCTO, a HIPAA-compliant web-application, as part of our participant tracking database. The SurveyCTO database will include participant names, phone numbers, addresses, and landmarks to further clarify one’s home location. The SurveyCTO interface will create a record for each study participant, which will then be linked to a family record, allowing for increased tracking of participant data. Prior to completing the post-assessments, RAs will update record forms for all participants to ensure all contact information is up to date. Ten days prior to the scheduled 12-month follow-up assessment, RAs will make calls reminding participants about their scheduled assessment. Missed calls will be noted and attempts to contact the participant will continue until contact is achieved. For those who are not reached by phone, RAs will visit participant homes to locate missing participants, leaving messages with neighbors if no one is home. This will be the last procedure enacted to locate participants. |
| Data management | 19 | All participant surveys, qualitative interviews, audio recordings, transcripts, and consent forms will be kept in locked files or encrypted electronic devices accessed only by the research team. Tablet-collected survey data and smartphone audio recordings will be de-identified, stored on encrypted devices, uploaded via a WIFI hotspot onto encrypted and password-protected laptops and backed up to Box and an external secure server at Brown University. All tablet-based data will be remotely wiped from the tablet once it has been uploaded to a cloud-based server and stored as encrypted files on password protected computers. Participant contact information, which is linked to participants, will be stored in SurveyCTO and kept separately from raw participant data.  Respondents’ names will not appear on the surveys or qualitative transcripts. Each survey or transcript will be labeled with a unique identifying code that renders the data anonymous. The master list, linking code numbers with names, will be stored separately in SurveyCTO. Computer data files will not include any identifying names. All data will be stored on a computer drive that is only accessible to members of the research team. Investigators will download data from this secure database for analysis. |
| Statistical methods | 20a | We will compare fidelity trajectories of teachers receiving mobile supervision to those of teachers receiving standard supervision. We will conduct exploratory analyses to uncover typical trajectories for each supervision method (mobile-based or standard) and undertake comparisons using growth curve modeling. We will investigate the null hypothesis that mobile supervision will not differ from standard supervision, either in fidelity trajectories across the 12 sessions or at post-intervention in student outcomes. To investigate YRI effects on mental health at post-intervention, we will use multilevel models to accommodate the clustering of students within YRI groups.  We will examine fidelity as an implementation outcome by comparing fidelity trajectories in standard vs. mobile supervision. Exploratory analyses (e.g., graphical) will identify typical trajectories for each supervision method and compare trajectories using growth curve modeling.  A mixed methods approach will be used to examine implementation level outcomes. Qualitative data will be analyzed by two trained coders using rapid qualitative analyses methods for implementation science research. Qualitative and quantitative data will be synthesized and triangulated to identify barriers and facilitators to YRI implementation and factors influencing adoption and sustainment of the YRI within schools. We will analyze qualitative and quantitative findings using “joint displays” to identify areas of synergy. For example, qualitative data on feasibility, acceptability and readiness to change might sync with quantitative data on YRI fidelity. |
|  | 20b | We do not expect to find a significant school-level intraclass correlation for targeted YRI outcomes, and if this is the case, then a two-level model will be estimated with sector and urban/rural estimated in the group-level equation. We intend to explore the role of student demographic characteristics and session attendance at the student level. |
|  | 20c | Multilevel modelling will handle missing data using multiple imputations based on the dependencies of multilevel data. Missing time points will be addressed by the multilevel approach to growth modelling, and data from all enrolled participants can be analyzed in accordance with our intention to treat design. |

| **Methods: Monitoring** | | |
| --- | --- | --- |
| Data monitoring | 21a | This study does not have a data safety and monitoring board because it was considered to pose minimal risk of harm to participants by the trail funders and sponsors. |
|  | 21b | Interim analyses are not planned for this study at the current time. Data cleaning procedures will be performed after each data collection time period. |
| Harms | 22 | Any adverse events that are observed and/or reported during assessments or intervention sessions will be reported immediately to the PI. The PI will receive these reports on an event-by-event basis. They will also be elicited in an open-ended manner through regular informal contact between the PI and the research staff. All adverse events will be reported in writing to the IRB within one week. All serious adverse events will be reported to the IRB immediately by telephone and by written report within 24 hours of our receipt of information regarding the event. The SAE will be reported to NIH within 48 hrs. At any time during the study period, if significant substance use or mental health concerns are reported, the participant will be referred to appropriate resources. Occurrences of adverse events would warrant formal review of the study procedures by the investigators with the aim of preventing further adverse events. The Principal Investigator will work closely with the IRB and the Office of Human Research Protections (OHRP) in this regard. |
| Auditing | 23 | Weekly data integrity checks will be performed by study RAs. |

| Ethics and dissemination | | |
| --- | --- | --- |
| Research ethics approval | 24 | This study has been reviewed and approved by the Brown University IRB and the Sierra Leone Ethics and Scientific Review Committee. |
| Protocol amendments | 25 | Any modifications to the protocol which may impact on the conduct of the study, potential benefit of the patient or may affect patient safety, including changes of study objectives, study design, patient population, sample sizes, study procedures, or significant administrative aspects will require a formal amendment to the protocol. Protocol amendments will be submitted to the Brown University IRB and the SLESRC for review and approval. The PI will notify the research team about all amendments to the protocol and provide documentation, which will be stored in the HIPAA compliant, secure Box folder. |
| Consent or assent | 26a | Study RAs will perform informed consent procedures for study participants. All study RAs will complete CITI training in human subjects research and best clinical practices. All study participants will be provided a copy of the informed consent form, and study RAs will read the form aloud together with potential participants to ensure comprehension. The consent will describe the purpose of the study, the participant's involvement, where the study will be conducted, how much time participation is expected to entail, and the information they will be asked to provide. Participants will also be told that participation is voluntary and they are free to withdraw participation at any time. Sufficient time will be allowed for questions about the consent forms or about the study in general. For minor youth (aged 14-17), we will obtain parental consent and youth assent. RAs will schedule parental consent sessions to explain the purpose of study, duration of the study, and potential risks and benefits of participation, after which RAs will hold youth assent sessions. |
|  | 26b | N/A |
| Confidentiality | 27 | An assigned study ID code will link all sources of information; only the Principal Investigator and study team members will have access to identifying information. We will encrypt and password protect all tablets using a password known to the research staff and Program Manager. All data will remain on the tablet until it is connected to WIFI and uploaded to a secure server. Daily quality assurance and data monitoring checks will determine successful upload of the data before it is remotely wiped from the tablet. Data will be backed up to Box, a secure, HIPAA-compliant, cloud-based storage platform. We will instruct teachers and supervisors that in the event of loss or theft, they must immediately notify the PIs with the tablet serial number to initiate a remote wipe of the tablet by IT.  Teachers and YRI supervisors will also receive training on protocols for information sharing via the mHealth app and WhatsApp to ensure that all communication during supervision chats is HIPAA compliant. The most current WhatsApp privacy policies ensure end-to-end encryption for personal, peer-to-peer, messages. During YRI delivery, teachers will use study-provided smart phones to audiotape sessions and receive training in protocols to securely upload encrypted data to the HIPAA compliant, cloud-based storage platform. |
| Declaration of interests | 28 | No competing interests to declare. |
| Access to data | 29 | All study investigators will have access to the final trial data set. Other The PI and her collaborators are aware of and agree to abide by the principles for sharing research resources, as described by NIH in NIH Data Sharing Policy and Implementation Guidance and more specifically in “Data Sharing Expectations for NIMH-funded Clinical Trials” and in any other applicable NIH sharing policies and related guidance. The trial data will be entered into the NIMH Data Archive as required and prescribed by the Notice of Award. |
| Ancillary and post-trial care | 30 | Harm related to trial participations is anticipated to be rare and risk of harm is minimal. In the event that a participant reports social harm or psychological distress resulting from study participation, the study social worker will make referrals to appropriate resources for the safety of the participant, as needed. This includes services for housing and food insecurity, domestic violence, mental health, and social services. Information regarding potential social harm will be recorded systematically in study logs. All instances of immediate risk of harm (e.g., situations involving risk of harm to self or others) will activate the study safety plan. This will involve intervention from the study psychologist and research staff, and participation in the study will stop. The PIs will also be notified in all such instances. Serious risk of harm cases will be well documented and reported to the IRB, and appropriate referrals will be enacted. If necessary and with the consent of participants, a trusted community or family member may also be enlisted as a source of interpersonal support. |
| Dissemination policy | 31a | Data sharing will be an important part of this project because resulting data could inform decisions of stakeholders and policy makers on implementation of mHealth interventions in Sierra Leone and other Low- and Middle- Income Countries. The study leaders are deeply committed to making the findings available to researchers in the field, as well as community leaders and government and non-government stakeholders. The Program Manager will lead development of policy briefs, newspaper articles, radio announcements, and social-media site postings of study findings to target multiple audiences through multiple modalities, including key stakeholders and policy makers (e.g., Ministry of Basic and Senior Secondary Education, Ministry of Youth Affairs, DSTI, World Bank). Brown University will comply with NIMH policy regarding submission to the NIMH Data Archive for clinical trial data. We plan to attend and present the results of our work at key scientific conferences when possible. We will also endeavor to present our findings at regional meetings that may be held in Africa. All final peer-reviewed manuscripts that arise from this proposal will be submitted to the digital archive, PubMed Central. There are a wide number of potential publication avenues for results stemming from the proposed project. |
|  | 31b | Authorship eligibility includes involvement in at least one of the following: the conceptualization and design of the study, implementation of study activities and collection of data, analysis and interpretation of data; and also in the writing and revising of manuscript and review for final publication. Professional writers will not be used. |
|  | 31c | In response to requests to share research materials and tools, the Uniform Biological Material Transfer Agreement and a revised version of that agreement for non-biological materials will be used. Those agreements typically cover rights to derivatives fairly and equitably. Depending on the nature of the data or materials involved, Brown University may use Non-Disclosure Agreements in order to protect intellectual property rights. The purpose of such agreements is to enable sharing of data and materials for the benefit of the scientific community while protecting the legitimate intellectual property rights belonging to Brown University and their respective researchers. The PI and her collaborators are aware of and agree to abide by the principles for sharing research resources and data, as described by NIH in NIH Data Sharing Policy and Implementation Guidance and more specifically in “Data Sharing Expectations for NIMH-funded Clinical Trials” and in any other applicable NIH sharing policies and related guidance. The data generated in this clinical will be entered into the NIMH Data Archive as required and prescribed by the Notice of Award as well as presented at national and/or international conferences and published in a timely fashion. All final peer-reviewed manuscripts that arise from this proposal will be submitted to the digital archive PubMed Central. Published data will be available in print or electronically from publishers, subject to subscription or printing charges. Research data that document, support and validate research findings will be made available after the main findings from the final research data set have been accepted for publication. We are committed to providing full access to all behavioral data and are actively searching for the most appropriate vehicle to do so. |
| Appendices |  |  |
| Informed consent materials | 32 | Sample consent forms can be provided upon specific request. |
| Biological specimens | 33 | N/A |
